# Trunk Muscle Activity Is Modified in Osteoporotic Vertebral Fracture and Thoracic Kyphosis with Potential Consequences for Vertebral Health

**DOI:** 10.1371/journal.pone.0109515

**Published:** 2014-10-06

**Authors:** Alison M. Greig, Andrew M. Briggs, Kim L. Bennell, Paul W. Hodges

**Affiliations:** 1 Department of Physical Therapy, University of British Columbia, Vancouver, Canada; 2 School of Physiotherapy and Exercise Science, Curtin University, Perth, Australia; 3 Arthritis and Osteoporosis Victoria, Melbourne, Australia; 4 Centre for Health, Exercise and Sports Medicine, The University of Melbourne, Melbourne, Australia; 5 The University of Queensland, Centre for Clinical Research Excellence in Spinal Pain, Injury and Health, School of Health and Rehabilitation Sciences, Brisbane, Australia; Faculté de médecine de Nantes, France

## Abstract

This study explored inter-relationships between vertebral fracture, thoracic kyphosis and trunk muscle control in elderly people with osteoporosis. Osteoporotic vertebral fractures are associated with increased risk of further vertebral fractures; but underlying mechanisms remain unclear. Several factors may explain this association, including changes in postural alignment (thoracic kyphosis) and altered trunk muscle contraction patterns. Both factors may increase risk of further fracture because of increased vertebral loading and impaired balance, which may increase falls risk. This study compared postural adjustments in 24 individuals with osteoporosis with and without vertebral fracture and with varying degrees of thoracic kyphosis. Trunk muscle electromyographic activity (EMG) associated with voluntary arm movements was recorded and compared between individuals with and without vertebral fracture, and between those with low and high thoracic kyphosis. Overall, elderly participants in the study demonstrated co-contraction of the trunk flexor and extensor muscles during forwards arm movements, but those with vertebral fractures demonstrated a more pronounced co-contraction than those without fracture. Individuals with high thoracic kyphosis demonstrated more pronounced alternating flexor and extensor EMG bursts than those with less kyphosis. Co-contraction of trunk flexor and extensor muscles in older individuals contrasts the alternating bursts of antagonist muscle activity in previous studies of young individuals. This may have several consequences, including altered balance efficacy and the potential for increased compressive loads through the spine. Both of these outcomes may have consequences in a population with fragile vertebrae who are susceptible to fracture.

## Introduction

Osteoporosis is a significant public health problem particularly in women, with vertebral fractures recognised as one of the hallmarks of the condition. The “vertebral fracture cascade” refers to the 4–7 fold increased risk of subsequent vertebral fractures and increased risk of fracture in the appendicular skeleton (e.g. odds ratio of 2.8 for developing a fracture at the femoral neck [1]), after an incident fracture is sustained [2]. Physical impairments, psychosocial morbidity and health care costs increase similarly as the frequency of fractures increase [3], [4]. Identification of individuals at risk of sustaining an incident vertebral fracture, or recurrent fractures, is a priority.

Low bone mineral density alone is an unreliable predictor of vertebral fracture at the individual patient level [5]. This suggests other factors moderate fracture risk. A comprehensive review identified several non-osseous factors as potential contributors to the fracture cascade. These include neurophysiologic properties such as trunk muscle activation (which may increase spinal load) and compromised balance (which may increase falls risk) [2]. Despite limited research, some data suggest balance impairments in populations with osteoporosis, particularly those with greater thoracic kyphosis [6], [7]. However, as the presence of existing vertebral fractures was not investigated in the osteoporotic populations in these studies, it is not possible to determine whether balance deficits were mediated by postural change or the presence of fractures. Our recent study showed that balance impairment is related to vertebral fracture rather than thoracic kyphosis among women with osteoporosis [8]. A further complication for investigation of factors that contribute to fracture risk is the differentiation between the presence of a vertebral fracture and normal age-related changes in vertebral morphology. It is necessary to disentangle the relative contribution of thoracic kyphosis and fracture to functional changes associated with osteoporosis to guide appropriate selection of interventions.

Changes in trunk muscle activation could underlie both the increased fracture risk and balance deficits. Preliminary evidence shows greater activation of paraspinal (extensor) muscles, which increase spinal load, in individuals with vertebral fractures than those without [9]. From other populations, decreased spinal mobility related to increased back muscle activation has been linked to compromised balance [10], [11]. However, several questions remain unanswered. First, activity of other trunk muscles remains unexplored in people with osteoporosis, and if co-activation of trunk flexor and extensor muscles is increased this substantially increases spinal load, as is common in back pain [12]. Second, changes in trunk muscle activity could be related to the degree of thoracic kyphosis or the presence of vertebral fracture [8], and these have not been differentiated.

Investigation of postural adjustments is an ideal model to investigate changes in trunk muscle activation in osteoporosis. Analysis of anticipatory postural adjustments in association with arm movements [13]–[15] provides an opportunity to investigate the pre-programmed strategy initiated by the nervous system to counteract predictable challenges to balance. Anticipatory postural adjustments are affected by a number of factors that are associated with osteoporosis and fracture, including posture [16], pain [17]–[19], and fear of falling [20]; these latter two effects are known to be mediated by increased co-contraction of antagonist flexor and extensor trunk muscles during the postural adjustments [20], [21].

This study aimed to investigate differences in activity of the trunk muscles using electromyography (EMG) during postural adjustments in older individuals with primary osteoporosis. Participants were divided into groups based on; (i) the presence or absence of osteoporotic vertebral fractures, and (ii) the magnitude of thoracic kyphosis (high vs. low).

## Materials and Methods

### Participants

Twenty-four community-dwelling women with primary osteoporosis and more than five years post menopause were recruited for this study. Women were recruited from the community via local advertising (newspapers, posters) and by approaching osteoporosis peer support groups in metropolitan Melbourne. Participants were also recruited from local private medical specialist clinics and public outpatient clinics and bone densitometry units at the Royal Melbourne Hospital and Broadmeadows Hospital.

Subject to meeting the inclusion criteria (diagnosis of primary osteoporosis, pain <4/10, age ≥55 years and more than 5 years post menopause), participants were divided into fracture (n = 10), and no-fracture (n = 14) groups. Participants were also independently grouped based on low (n = 12) and high (n = 12) thoracic kyphosis. Exclusion criteria included any other medical conditions that affect bone metabolism or balance, or participation in any high intensity exercise that could affect trunk control. Physical activity was assessed using the Physical Activity Scale for the Elderly (PASE) [22]. Pain immediately prior to and during testing, was assessed using an 11-point numerical rating scale (NRS) anchored with “no pain” and “worst pain imaginable”. Pain of less than 4/10 was required for participation in this study in order to ensure comfort and safety of participants and overcome the potentially moderating influence of pain on EMG responses.

Osteoporosis was diagnosed from bone densitometry results using World Health Organization criteria [23], and vertebral fractures were diagnosed from standardised lateral radiographs (lumbar and thoracic spine) using conservative classification criteria [24] as reduction in anterior vertebral height of ≥30% compared to posterior height and the posterior height of the adjacent superior or inferior vertebral body. Compression fractures were identified from qualitative reviews of spinal radiographs, consistent with an accepted semi-quantitative method [25]. Lateral spine radiographs were used to measure thoracic kyphosis using the vertebral centroid angle (T4 to T9). Reliability and validity for the centroid measure have been established, and this measure is considered superior to traditional methods such as the Cobb angle [26], [27]. Nonetheless, traditional Cobb angles (T 4–9) were also measured according to Goh and colleagues [28], for comparison to previous literature.

**Figure 1:**
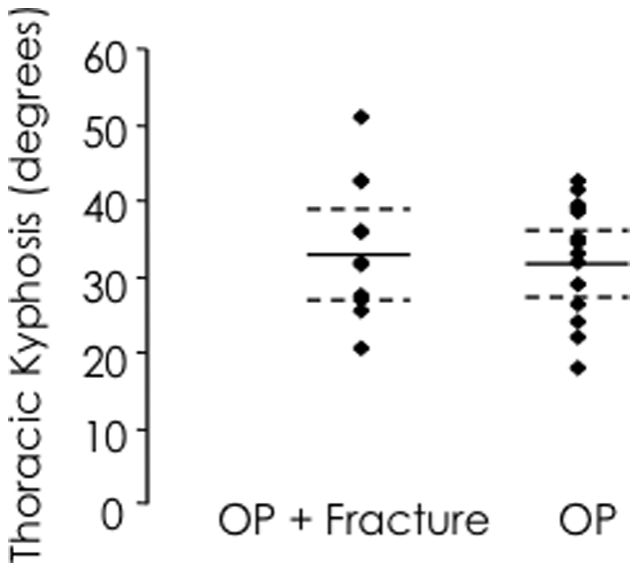
Magnitude of thoracic kyphosis based on fracture group. Mean and 95% confidence interval for each group (fracture and no-fracture) is shown. Thoracic kyphosis was not significantly different between groups (*p* = 0.660).

Participants were grouped into two categories based on the: i) presence of vertebral fracture; and ii) degree of thoracic kyphosis. For the first analysis, based on fracture, there were no differences in demographic characteristics between groups categorised in this manner (all *p*>0.092) (Table 1). Seventeen anterior wedge fractures were identified in the fracture group at vertebral levels T4 (17.6%), T5 (11.8%), T6 (23.5%), T7 (11.8%), T8 (23.5%), T9 (5.9%) and T12 (5.9%). Thoracic kyphosis was not different between fracture groups based on either the centroid (*p* = 0.660) or Cobb (*p* = 0.125) measurements (Fig. 1). For the second analysis participants were divided by the median centroid angle of 35°in to low and high thoracic kyphosis groups. We elected to divide the group into these high and low categories using a median split in the data rather than using clinical thresholds for thoracic hyper-kyphosis (determined by traditional Cobb-based measurement approaches), as the Cobb method is particularly prone to measurement error in populations with osteoporosis. For example, the Cobb angle predominantly reflects endplate tilt of vertebrae between selected limits of the curve, and may not reveal changes regionally within the curve, nor true intervertebral curvature relative to vertical [26], [27]. Although the vertebral centroid method has been used previously in a population with osteoporosis and measurement properties established [26], there is currently no agreement regarding a threshold for classification of hyper-kyphosis. Although the median-split approach arbitrarily categorized participants into “high” and “low” groups, it enabled valid statistical comparison between equal-sized groups and comparable variance based on visual inspection of the spread of the data. When considering the traditional Cobb angle method for measuring thoracic kyphosis, we observed a Cobb angle between T4-9 of greater than 40° (hyper-kyphosis) in 12 (50%) participants. There were no differences in demographic parameters between kyphosis groups (all *p*>0.103) (Table 2). Pain was <2/10 for all participants during testing and was not different between groups (*p*>0.05). Ethical approval was granted by the Institutional Human Research Ethics Committee and all participants provided written, informed consent.

**Table 1:** Descriptive statistics for fracture groups expressed as mean (SD).

	Fracture (n = 10)	No Fracture (n = 14)	
Height (cm)	158.9 (5.4)	157.5 (4.1)	*p* = 0.486
Mass (kg)	67.0 (10.8)	59.8 (9.2)	*p* = 0.092
Age (years)	68.1 (7.1)	64.0 (8.9)	*p* = 0.239
BMI (kg/m^2^)	26.6 (4.5)	24.5 (3.8)	*p* = 0.235
Kyphosis - Cent 4–9 (deg)	34.1 (9.4)	32.5 (7.6)	*p* = 0.660
Kyphosis - Cobb 4–9 (deg)	44.5 (9.8)	38.5 (8.4)	*p* = 0.125
PASE†	164.4 (50.6)	156.7 (54.5)	*p* = 0.738

† Physical Activity Scale for the Elderly.

**Table 2:** Descriptive statistics for thoracic kyphosis groups expressed as mean (SD).

	Low Kyphosis (n = 12)	High Kyphosis (n = 12)	
Height (cm)	157.9 (5.3)	158.2 (4.0)	*p* = 0.905
Mass (kg)	59.3 (10.3)	66.2 (9.6)	*p* = 0.103
Age (years)	64.8 (6.9)	66.6 (9.7)	*p* = 0.615
BMI (kg/m^2^)	24.3 (4.4)	26.5 (3.8)	*p* = 0.203
Kyphosis - Cent 4–9 (deg)	26.4 (4.7)	39.9 (4.6)	p<0.001^a^
Kyphosis - Cobb 4–9 (deg)	35.1 (6.5)	46.8 (8.0)	p<0.001^a^
PASE†	154.0 (47.1)	166.0 (58.5)	p = 0.591

a significant difference.

† Physical Activity Scale for the Elderly.

### Electromyography

Electromyographic activity (EMG) was recorded using pairs of Ag/AgCl adhesive electrodes (1 cm discs, Meditrace, Kendall LTP, MA, USA), placed along the muscle (inter-electrode distance - 2 cm). Recordings were made of obliquus internus (OI), and externus abdominis (OE), rectus abdominis (RA), and erector spinae (ES) at L3 and T7 EMG [29], [30]. EMG electrodes were placed over the anterior and posterior deltoid, and a ground electrode was placed over the right iliac crest. EMG data were amplified 1000x, band pass filtered between 20–1000 Hz (2^nd^ order Butterworth 12dB/octave filter) and sampled at 2000 Hz. A notch filter was used at 50 Hz. Data were recorded and stored using a Power1401 data acquisition system and Spike 2 (v 4.10) software (Cambridge Electronic Design Limited, Cambridge, England), and exported for analysis with Matlab 6.5.0 (The Mathworks, Natick, MA, USA).

### Task Protocol – Anticipatory Postural Adjustments

Participants rapidly moved their right arm to disturb balance while standing with feet shoulder-width apart, toes aligned forward, and equal weight through both feet. Participants move their arm forward (shoulder flexion to ∼60°) or backwards (shoulder extension to ∼40°) as rapidly as they could in response to a light that indicated the direction of movement. Participants were instructed to relax between arm movements. The light was triggered manually and recorded with EMG data. Direction of arm movements was randomized for 10 trials in each direction (forward or backward), resulting in a total of 20 unique arm movements performed in a random sequence. An accelerometer (Crossbow Technology Inc, San Jose, CA, USA) was fixed to the dorsum of the right hand to provide information regarding movement onset and arm displacement. Five practice trials were undertaken with each participant to ensure they understood the protocol.

### Data Analysis

Deltoid EMG provided information about the direction and timing of arm movements to which trunk EMG could be related. The time of onset of deltoid EMG was identified visually as this method has been shown to be reliable and less affected by background EMG than many statistical based methods [31]. We elected to characterise the pattern of the EMG activity of the trunk muscles based on analysis the activity of each muscle, averaged over participants, within epochs/time intervals either side of the onset of deltoid EMG rather than analysis of the onsets of EMG of each of the trunk muscles. The main reason underpinning this decision was the difficulty in to identify the onset of EMG, particularly of the thoracic extensor muscles, when there is ongoing tonic activity. This approach has been successfully used to characterise postural patterns of muscle activation in earlier studies [32]. Root mean square (RMS) EMG amplitude was calculated for each trunk muscle in ten 50-ms epochs (from 250 ms before to 250 ms after deltoid EMG onset). Maximum voluntary contractions were not performed in this study for EMG normalisation due to risks associated with vertebral fragility. Normalisation to a submaximal task is also not appropriate as this would lead to inaccurate conclusions if the different groups perform the submaximal task differently. Due to these limitations, EMG data were analysed in two alternate ways. First, EMG data were normalised to the peak activity recorded for each muscle, across all epochs and trials. This approach to normalisation enables a valid comparison in EMG amplitude between epochs and between arm movement directions for a given muscle. Given this approach does not use a between-muscle normalisation, such as maximum voluntary contraction across muscles, the method precludes valid comparison of EMG amplitude between muscles and between participant groups. This approach to normalisation has been used previously [9]. Second, non-normalised EMG amplitude was used for comparison of each muscle between groups. This analysis is affected by differences in electrode placement and filtering by subcutaneous tissue. However, as there was no difference in BMI between fracture groups (*p* = 0.232) or groups based on thoracic kyphosis (*p* = 0.203) we had no reason to suspect any systematic difference in subcutaneous tissue between groups. We argue that, with some caution, interpretation using the non-normalised EMG data could be made for an exploratory analysis in conjunction with the analysis of EMG pattern from data normalised to peak EMG.

### Statistical Analysis

Repeated measures analysis of variance (ANOVA) was used to compare normalised EMG amplitude between Epochs and arm movement Directions for each muscle. This analysis provided detailed information about the *pattern* of muscle activation and enabled identification of the epochs during which EMG activity increased above baseline (*epoch 0*). The pattern of trunk muscle activation was qualitatively compared between groups. In addition we undertook an exploratory analysis on non-normalised EMG data to compared EMG amplitude between groups for each muscle and each arm movement using independent t-tests. As this study was exploratory in nature, it was not considered appropriate to apply adjustments for multiple comparisons as this has been argued to mask potentially important differences [33]. Statistical analyses were conducted using SPSS for windows (v 11.0.1; SPSS Inc, Chicago, Illinois, USA) and significance was set at *p*<0.05.

## Results

### Electromyography during arm movement

**Figure 2:**
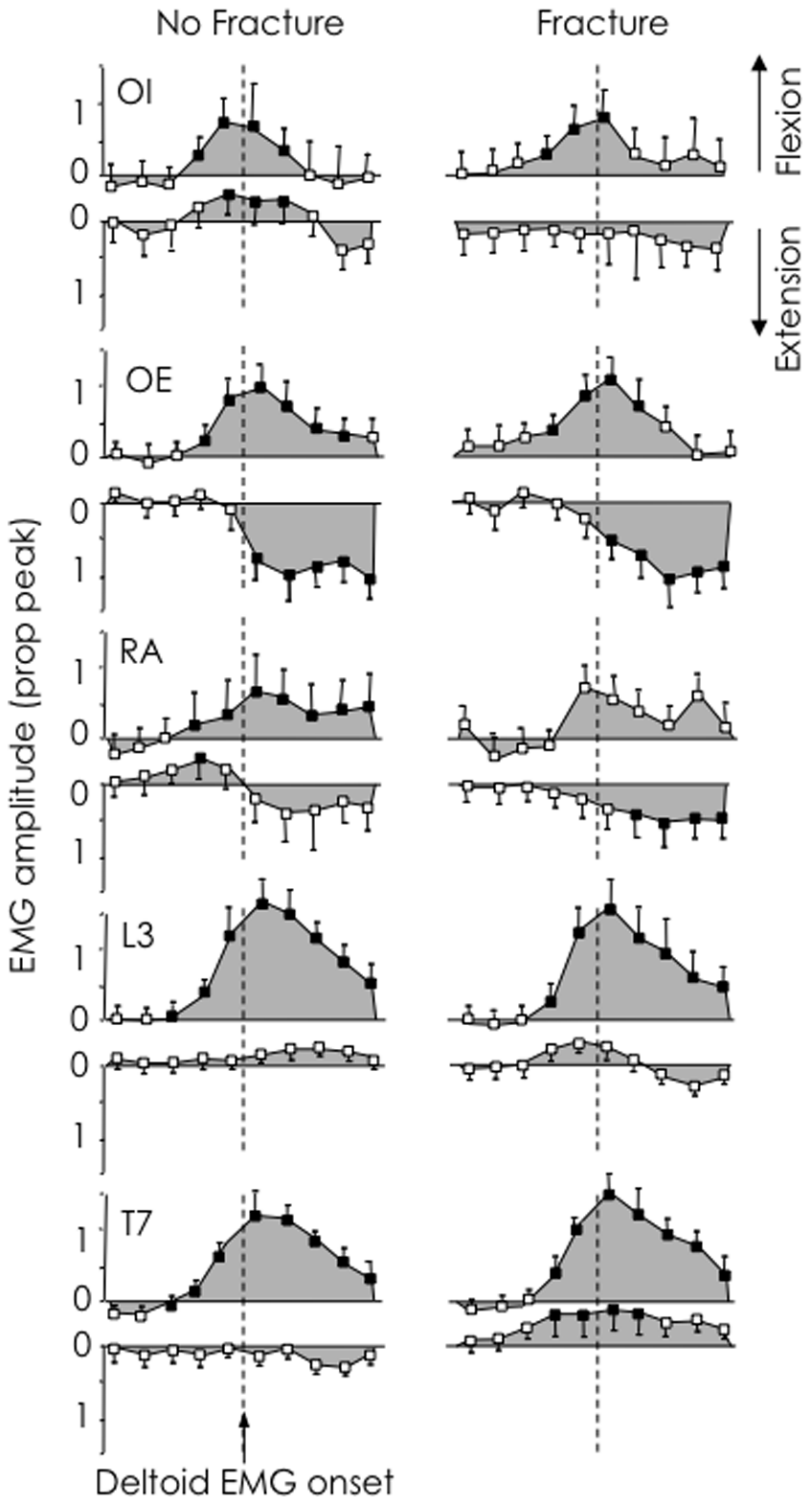
Average normalised EMG amplitude of trunk muscles for 10 epochs (250 ms before and after onset of deltoid EMG). Up-going panels and down-going panels demonstrate forwards and backwards arm movements, respectively. Filled shapes denote values that differ significantly (*p*<0.05) in amplitude from the baseline, and unfilled shapes denote values that are not different from baseline. Abdominal (OI, OE and RA) and back (Erector spinae at L3 and T7) increased during the same epoch during arm flexion rather than the predicted earlier onset of back muscle activity.

When participants moved their arm forwards, bursts of EMG activity of the trunk flexor and extensor muscles were initiated almost simultaneously. Such overlapping activity of antagonist muscles (up-going panels in Fig. 2) is consistent with a trunk co-contraction pattern. In general, during arm flexion, EMG amplitude increased above baseline (*p*<0.05, filled shapes in Fig. 2) during *epochs 3* to *5* (150-0 ms prior to deltoid) for both trunk flexor and extensor muscles.

Backwards movement of the arm was not accompanied by co-contraction of the trunk muscles. Instead, there was a burst of trunk flexor muscle EMG and either no change or decreased activity of the extensor muscles (down-going panels in Fig. 2). Activity of OE and RA increased above baseline (filled shapes in Fig. 2) during *epochs 6* to *7* (from deltoid onset, to 100 ms after).

### Association with fracture

Some differences in muscle activation were evident between fracture and no-fracture groups (Fig. 2). Irrespective of fracture status, EMG of OI and OE increased above baseline in epoch 4 (100–50 ms prior to deltoid onset). RA EMG only increased above baseline in the no-fracture group and this also occurred in epoch 4 (100–50 ms prior to deltoid onset). During forward arm movements ES EMG at L3 and T7 increased during a later epoch in the fracture group compared with no-fracture group (*epoch 4* vs. *3*).

**Figure 3:**
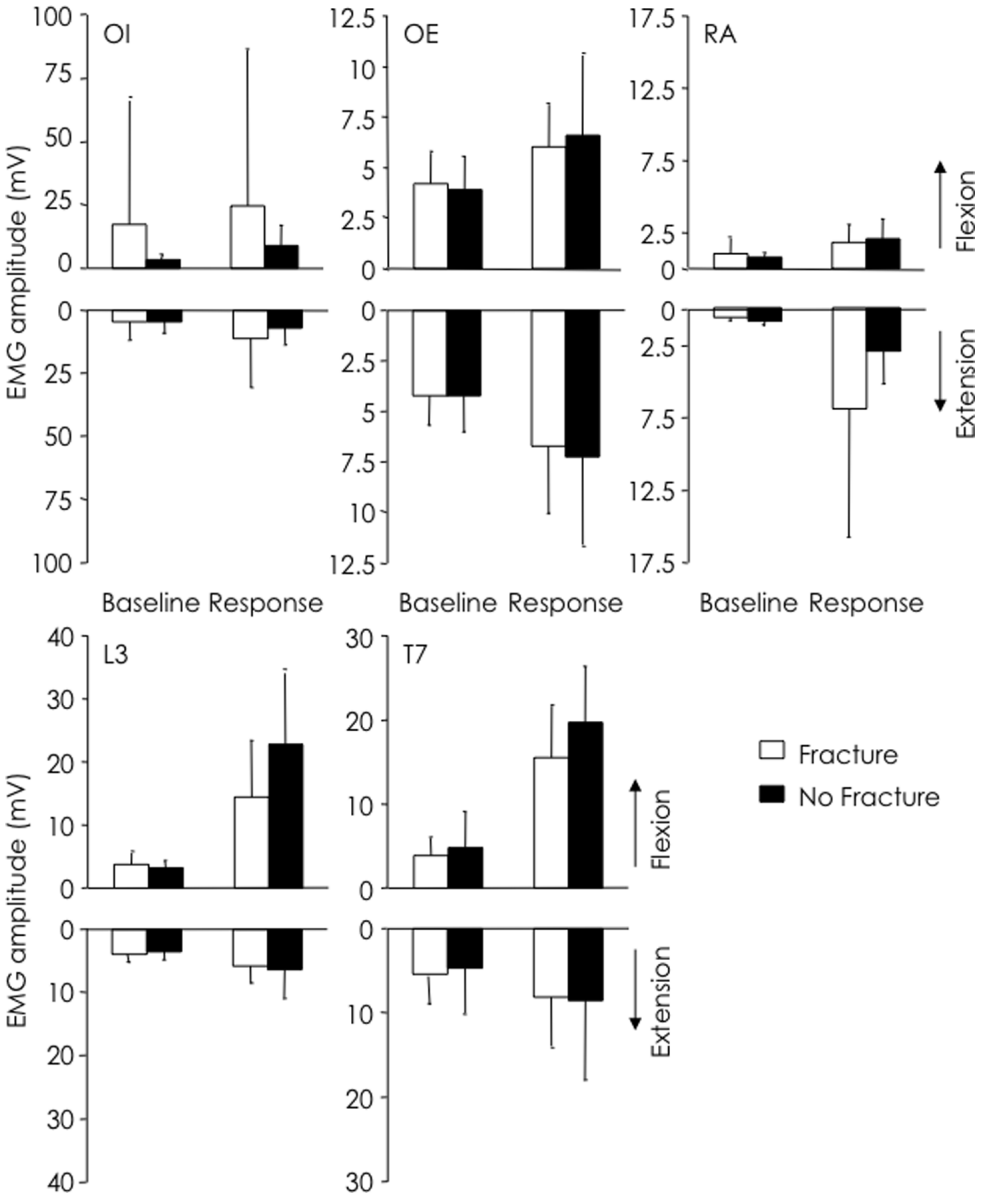
EMG amplitude based on fracture grouping. EMG amplitude at baseline (*epoch* 0) and during response (*epochs* 6–10) for fracture and no-fracture groups during forwards (up-going) and backwards (down-going) arm movements. There was no difference between groups for any muscle.

During backwards arm movements, OI EMG did not change from baseline in the fracture group, but reduced from baseline during *epochs 5* to *7* (50 ms prior to and 100 after deltoid onset) in the no-fracture group. RA EMG increased from baseline during *epochs 7* to *10* (100 to 250 ms after deltoid) in the fracture group, but there was a decrease in RA EMG in *epoch 4* in the no-fracture group. Activity of ES T7 decreased below baseline during *epochs 3* to *6* (150 ms prior to and 50 ms after deltoid onset) in the fracture group, while there was no change in ES T7 activity from baseline in the no-fracture group. Non-normalised EMG amplitudes was not different between fracture groups (Fig. 3; all p>0.116).

### Association with thoracic kyphosis

**Figure 4:**
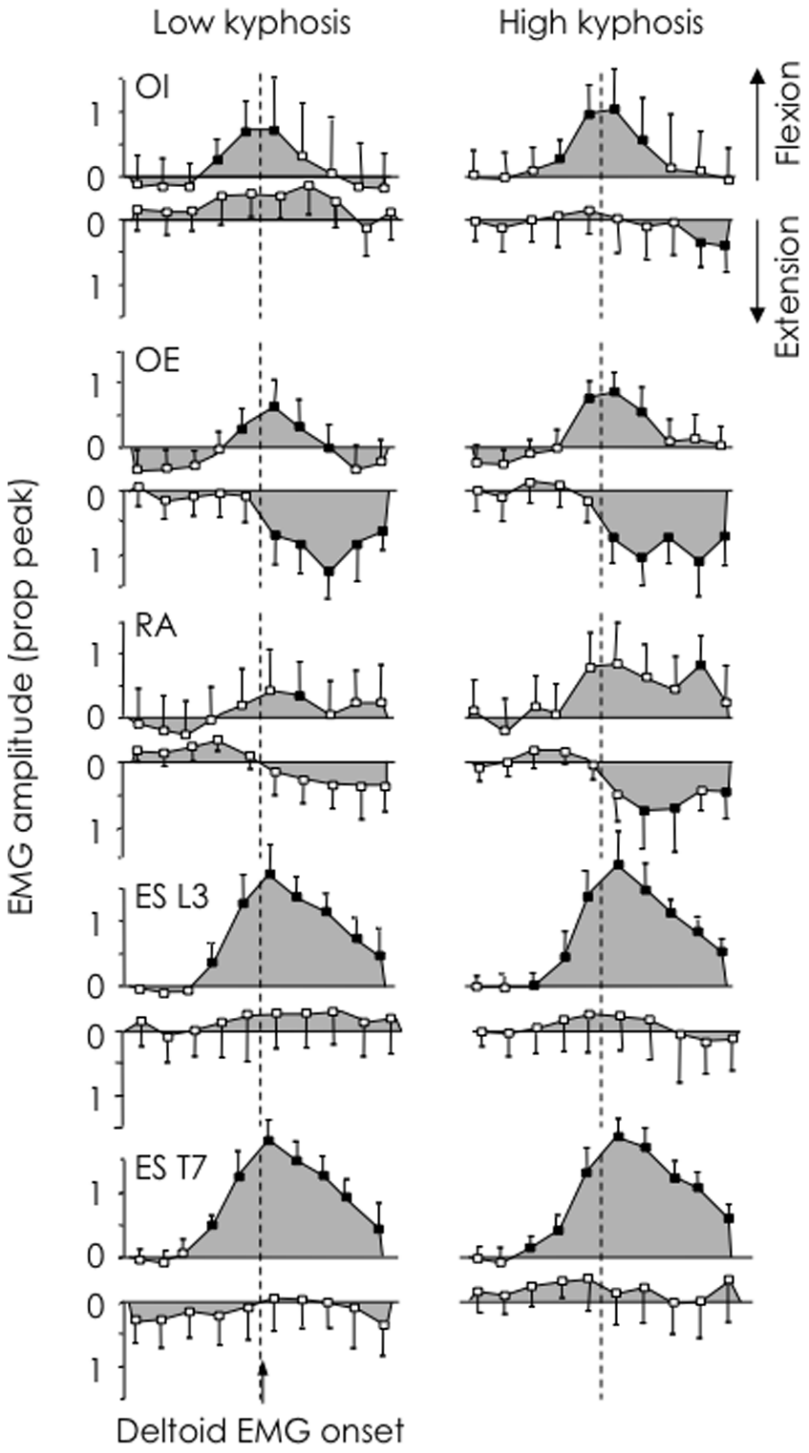
Average normalised EMG amplitude of trunk muscles for 10 epochs (250 ms before and after onset of deltoid EMG). Up-going panels and down-going panels demonstrate forwards and backwards arm movements, respectively. Filled shapes denote values that differ significantly (*p*<0.05) in amplitude from the baseline, and unfilled shapes denote values that are not different from baseline. Activity of back muscles (erector spinae at L3 and T7 increased earlier than flexors (OE and RA) during the forward arm movement.

When participants were grouped based on the magnitude of thoracic kyphosis, activity of trunk flexor muscles (up-going panels, Fig. 4) and extensor muscles (down-going panels, Fig. 4) showed a similar overlapping pattern, consistent with co-contraction. When participants in both groups moved their arm forwards, OI and OE EMG increased above baseline in *epochs 4* and *5*, respectively. ES L3 and T7 EMG increased during an earlier epoch in the high kyphosis group compared with low kyphosis group (*epoch 3* vs. *4*, respectively).

Backwards arm movements were associated with bursts of trunk flexor EMG activity. In the high kyphosis group OE and RA EMG increased above baseline in *epochs 6* and *7*, respectively, but there was no change in trunk extensor muscle EMG. In the group with low kyphosis, OE EMG activity also increased above baseline in *epoch 6*, but there were no other changes in EMG activity in either trunk flexor or extensor muscles.

**Figure 5:**
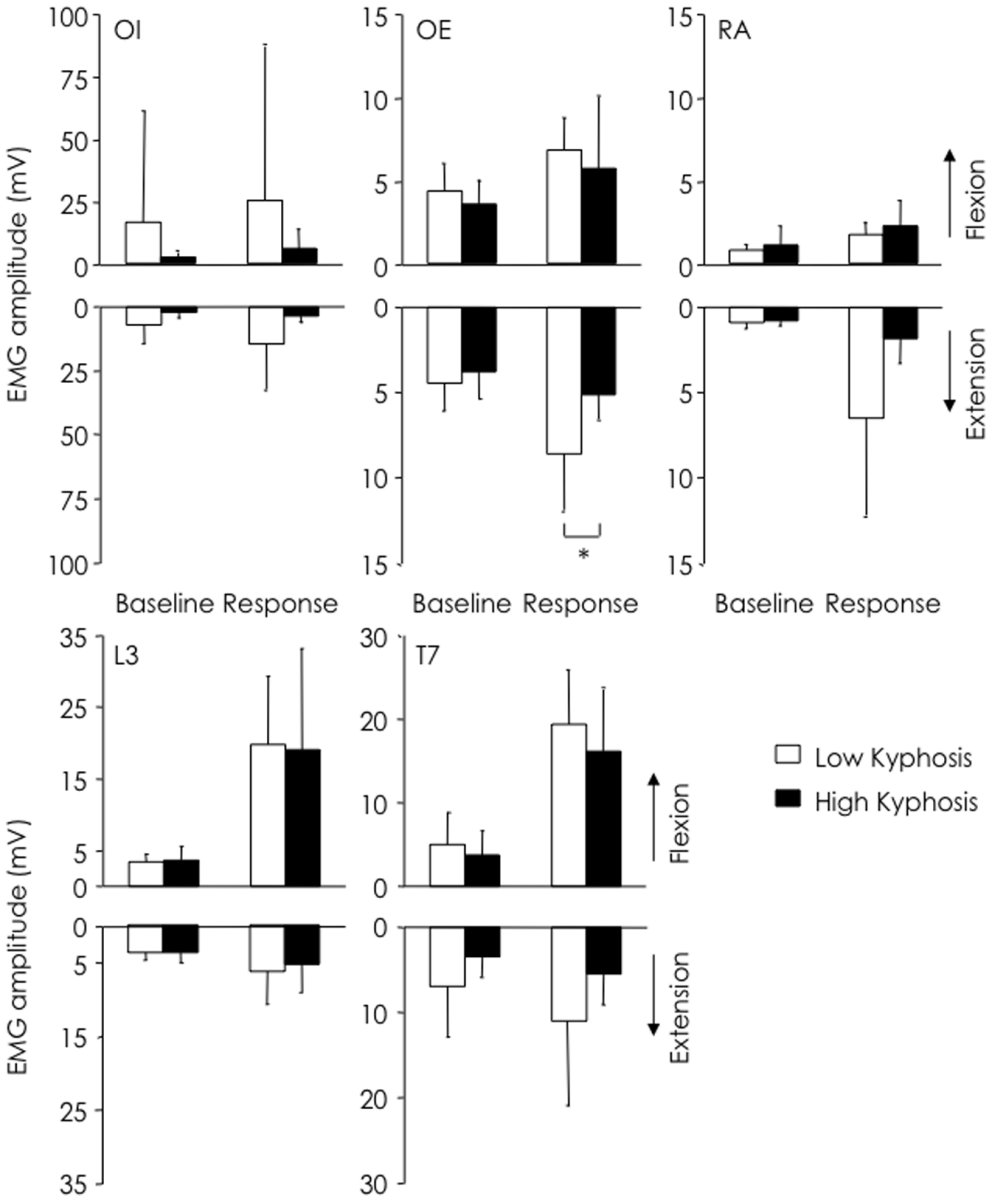
EMG amplitude based on kyphosis grouping. EMG amplitude at baseline (*epoch* 0) and during response (*epochs* 6–10) for thoracic kyphosis groups during forwards (up-going) and backwards (down-going) arm movements. Note significantly lower (*-P<0.05) OE EMG and a trend toward lower RA EMG in the group with least kyphosis.

Analysis of non-normalised EMG amplitude revealed less OE EMG in the group with high kyphosis, compared to low kyphosis (*p* = 0.029; Fig. 5). There were no other differences between groups for any muscle, but there was a non-significant trend toward lower RA EMG in the group with high kyphosis (*p* = 0.067).

## Discussion

The results of this study demonstrate that in this elderly population with established primary osteoporosis, unlike younger individuals reported previously [13], [34], arm flexion is associated with co-contraction of trunk flexor and extensor muscles. Here, we refer to our cohort as “elderly” as the majority of studies with comparable methods have sampled younger adults, and thus we refer to elderly in relative terms. Trunk co-contraction may have several consequences, including increased spinal compression and compromised postural recovery with implications for balance and falls risk. Both of these consequences may have negative implications in a population with increased vertebral fragility. Differences in trunk muscle activity were identified based on grouping by the presence of vertebral fracture or thoracic kyphosis. As there was no difference in thoracic kyphosis between the fracture groups in this cohort, influences of fracture and thoracic kyphosis appear to be independent.

### Co-contraction of the trunk antagonist muscles is increased in older individuals

A new observation in this elderly population was that antagonist trunk muscles co-contracted in association with voluntary forwards arm movements. This contrasts data from younger individuals that indicate a triphasic response of alternating antagonist trunk muscle activity to counteract the perturbation from arm movement [13], [32].

Alternating bursts of trunk muscle activity induces spine movement in advance of the arm movement [34]. Recent work in people with low back pain indicates reduced spine movement (which would be expected to accompany co-contraction) is associated with compromised quality of postural control [35]. Increased co-contraction of antagonist muscles (stiffening) in other body regions has also been reported in elderly populations. For example, ankle stiffness in standing is increased in the elderly population [36], and older individuals demonstrate longer durations of lower leg and trunk muscle co-contraction during external perturbations [37].

Stiffening strategies may be employed to maintain a tighter control of the body's centre of mass (COM) within the base of support [20], and to compensate for narrowed limits of stability associated with aging [38]. Thus, trunk stiffening may be an adaptive strategy of the neuromuscular system in the elderly. Given this study is cross-sectional in nature; we are unable to speculate conclusively on the drivers underpinning this trunk stiffening response. Although self-report physical activity data for our cohort suggest moderate to high levels of current physical activity as measured with the PASE instrument [22], we did not collect historic physical activity data to make inferences about how activity history and agility may influence the trunk motor control strategies. Nonetheless, the moderate to high levels of current physical activity point to our sample as active and imply that the trunk stiffening response is not secondary to inactivity and disuse which could impact the neuromotor system's strategies to maintain balance and control the trunk. Data from several studies suggest stiffening may be associated with fear of falling. Elderly individuals, who are less stable, demonstrate an increased tendency to stiffen their trunk in response to a tilting support surface [21], and lower leg stiffness is increased when moving to higher-threat conditions [20]. Although increased trunk stiffening may be an adaptation to improve postural control in static conditions, it may not be an optimal strategy when responding to perturbations of greater magnitude that require coordinated movement of the trunk to restore balance [15], [34]. Furthermore, increased loading from trunk muscle co-contraction [12], [39] may have negative consequences in individuals with spinal osteoporosis who have increased vertebral fragility. To our knowledge no physiologically-representative data are available which unequivocally identify the minimum forces required from trunk muscle co-activation to cause vertebral fracture, hence this suggestion remains speculative. Indeed, this may be an important area for future research.

Although co-contraction was increased in forwards arm movements across groups, this overlapping pattern of flexor and extensor muscle activity was not evident during backwards arm movements. This finding may be related to the argument that a forwards arm movements induces a riskier perturbation, as it creates a posterior displacement of the COM. Several authors argue posterior COM displacement induces greater falls risk because of compromised ability to generate ankle torque to counteract the COM displacement in that direction [40]. Taken together, co-contraction with forwards arm movements and evidence of increased stiffness in situations of high threat, suggest perturbation in this direction is more risky for the elderly individuals.

### Trunk muscle activity is altered in individuals with vertebral fracture

**Figure 6:**
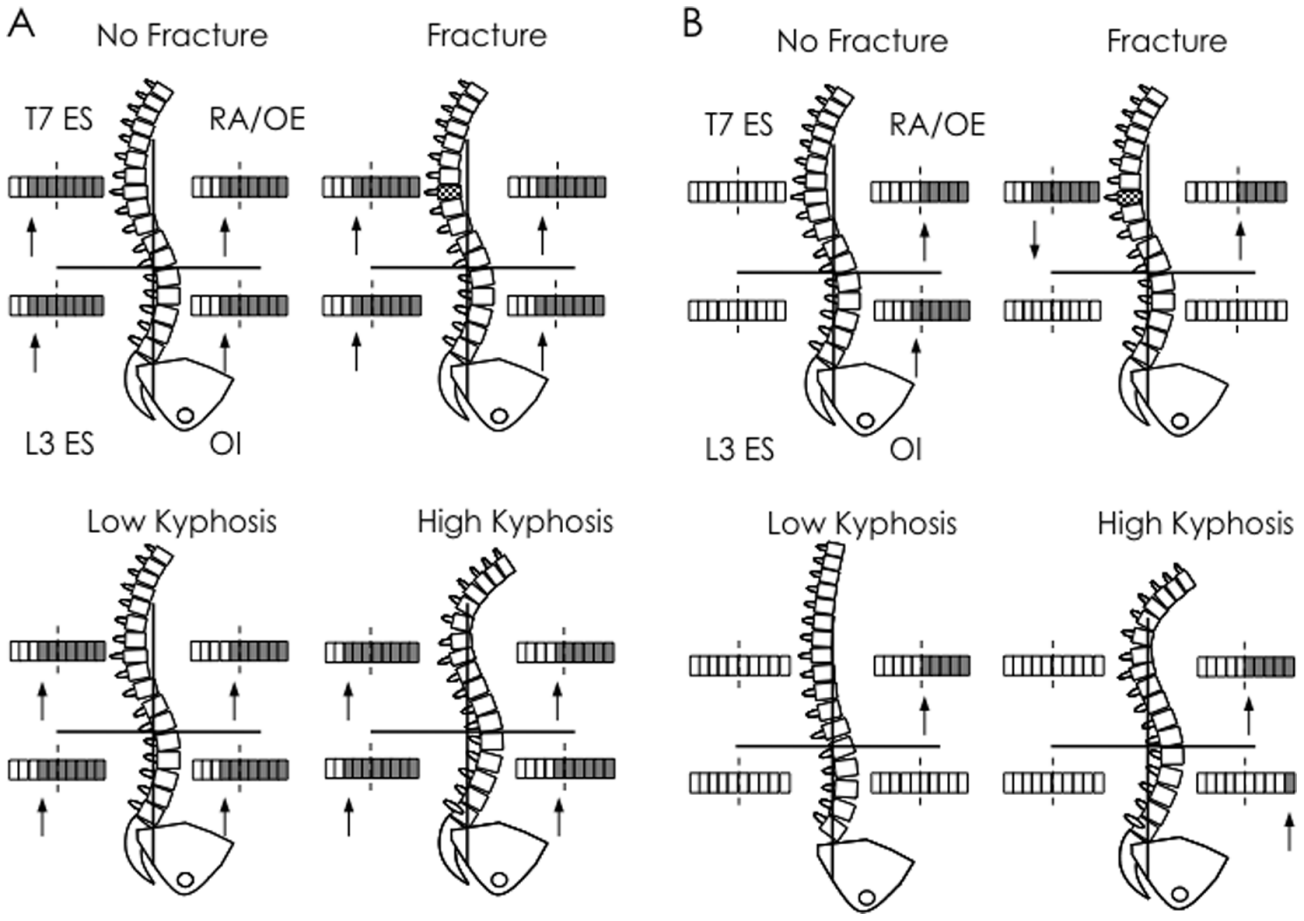
Summary of temporal differences in muscle activity in participants with osteoporosis grouped by presence or not of fracture and low and high kyphosis in (A) forwards and (B) backwards arm movements. Filled boxes indicate epoch in which muscle activity changes (“↑” increase and “↓” decrease). Boxes without fill indicate no change in activity from baseline.

In the present study, the participants with vertebral fracture demonstrated differences in trunk muscle activation compared with those without fracture (Fig. 6A). This finding agrees with a previous study of paraspinal muscle control in this population [9]. During forwards arm movements, individuals with fracture concurrently increased activity of both flexor and extensor muscles, whereas the no-fracture group activated trunk extensor muscles prior to trunk flexor muscles. The latter pattern is more representative of the triphasic response observed in young individuals [13], [34]. This finding highlights a more pronounced co-contraction strategy in individuals with fracture. The co-contraction response may indicate greater vertebral loading [12] during dynamic tasks. Taken together with increased vertebral loading observed in this population in a static situation [41], this may help explain, in part, the vertebral fracture cascade. The combination of increased static postural loading and the potential for further increases in loading during dynamic tasks may substantially increase vertebral fracture risk. Studies using EMG-driven biomechanical models would be required to clarify this issue.

During backwards arm movements the fracture group reduced paraspinal muscle activity at T7 to below the baseline amplitude (Fig. 6B). This reduction in back extensor muscle activity during a less risky perturbation direction may be an adaptation to reduce spinal compressive loads through fragile vertebral bodies, or reduce muscle activation around a previously painful fracture site. It is also possible that back extensor weakness, which is associated with fracture [42], may have forced individuals to use different trunk control strategies compared to the individuals without fracture.

### Trunk muscle activity is altered in individuals with greater thoracic kyphosis

When participants were grouped according to kyphosis angle the co-contraction strategy during forwards arm movements was evident in both low and high kyphosis groups (Fig. 6A). However, unlike the grouping based on fracture, the onsets of activity of the antagonist muscle groups were not simultaneous: trunk extensor EMG increased above baseline prior to arm movement, followed by trunk flexor muscles after arm movement onset. The most obvious differences between kyphosis groups was earlier onset of ES L3 and T7 in the group with high thoracic kyphosis and lower non-normalised OE EMG amplitude. These changes in the high kyphosis group may be explained by changes in mechanical demand for thorax control because of differences in posture. Increased forward spinal curvature and more anteriorly displaced COM from increased thoracic kyphosis places greater demand on the extensor muscles and reduced demand on flexors [7], [43]. The more anterior COM position also provides the main contribution to increased compression and shear at the spine in osteoporosis [41]. Prior to a forwards arm movement, the trunk extensor muscles create an extension moment and the COM moves posteriorly [44], [45]. In individuals with an already anteriorly displaced COM, the demand for extensor activity would be increased, and that of OE reduced. There were no marked differences in muscle activity recruitment patterns during backwards arm movements (Fig. 6B), but, variability in muscle activity within the groups may have obscured small differences.

### Methodological issues

Thoracic spine curvature did not differ between those with and without fracture using two radiographic measures of kyphosis (e.g. Cobb, centroid). This was surprising as thoracic curvature is expected to increase with fracture. One interpretation is that existing methods lack sensitivity to detect small differences in curvature, and thus alternative measurement approaches have been proposed [26].

The main EMG analysis involved normalisation to the peak EMG among trials. This provides a sensitive measure of pattern of activity and formed the primary analysis. In addition, we compared the non-normalised EMG amplitude. Although this has the potential for error due to differences in electrode placement and properties of subcutaneous tissues [46] between individuals, we argue the alternative methods of normalisation to maximal or submaximal efforts are either not possible in this group or introduce errors due to potential differences in strategy to perform submaximal tasks in a system with a complex array of muscles available to generate torque. The main risk from analysis of non-normalised data is lack of sensitivity to detect differences between individuals due to inherent variability. We believe the analysis of non-normalised data is justified and the identification of differences in amplitude between groups provides further context to interpret the changes in neuromuscular strategy as a secondary analysis.

### Conclusion

The results of this study demonstrate postural adjustments of trunk muscles activity with arm movements differ in elderly participants with osteoporosis and patterns of trunk muscle responses varied according to the presence of vertebral fracture and degree of thoracic kyphosis. The tendency to increase trunk muscle co-contraction in this elderly cohort may be detrimental for vertebral loading, especially in individuals with compromised bone strength. Further research is required to establish a temporal relationship between vertebral fracture and differences in trunk control strategies to determine cause and effect. In addition, future research is needed to evaluate whether rehabilitation of trunk control strategies to reduce reliance on trunk stiffening is clinically effective in prevention of subsequent vertebral fractures and falls.
